# Longitudinal transcriptomic analysis reveals persistent enrichment of iron homeostasis and erythrocyte function pathways in severe COVID-19 ARDS

**DOI:** 10.3389/fimmu.2024.1397629

**Published:** 2024-08-05

**Authors:** Moemen Eltobgy, Finny Johns, Daniela Farkas, Laura Leuenberger, Sarah P. Cohen, Kevin Ho, Sarah Karow, Gabrielle Swoope, Sonal Pannu, Jeffrey C. Horowitz, Rama K. Mallampalli, Joshua A. Englert, Joseph S. Bednash

**Affiliations:** ^1^ Department of Internal Medicine, Division of Pulmonary, Critical Care, and Sleep Medicine, The Ohio State University, Columbus, OH, United States; ^2^ Clinical Trials Management Office, College of Medicine, The Ohio State University, Columbus, OH, United States; ^3^ The Center for RNA Biology, College of Medicine, The Ohio State University, Columbus, OH, United States

**Keywords:** COVID - 19, ARDS (acute respiratory disease syndrome), RNA seq analysis, longitudinal analysis, SARS-CoV-2

## Abstract

**Introduction:**

The acute respiratory distress syndrome (ARDS) is a common complication of severe COVID-19 and contributes to patient morbidity and mortality. ARDS is a heterogeneous syndrome caused by various insults, and results in acute hypoxemic respiratory failure. Patients with ARDS from COVID-19 may represent a subgroup of ARDS patients with distinct molecular profiles that drive disease outcomes. Here, we hypothesized that longitudinal transcriptomic analysis may identify distinct dynamic pathobiological pathways during COVID-19 ARDS.

**Methods:**

We identified a patient cohort from an existing ICU biorepository and established three groups for comparison: 1) patients with COVID-19 ARDS that survived hospitalization (COVID survivors, n = 4), 2) patients with COVID-19 ARDS that did not survive hospitalization (COVID non-survivors, n = 5), and 3) patients with ARDS from other causes as a control group (ARDS controls, n = 4). RNA was isolated from peripheral blood mononuclear cells (PBMCs) at 4 time points (Days 1, 3, 7, and 10 following ICU admission) and analyzed by bulk RNA sequencing.

**Results:**

We first compared transcriptomes between groups at individual timepoints and observed significant heterogeneity in differentially expressed genes (DEGs). Next, we utilized the likelihood ratio test to identify genes that exhibit different patterns of change over time between the 3 groups and identified 341 DEGs across time, including hemoglobin subunit alpha 2 (*HBA1, HBA2*), hemoglobin subunit beta (*HBB*), von Willebrand factor C and EGF domains (*VWCE*), and carbonic anhydrase 1 (*CA1*), which all demonstrated persistent upregulation in the COVID non-survivors compared to COVID survivors. Of the 341 DEGs, 314 demonstrated a similar pattern of persistent increased gene expression in COVID non-survivors compared to survivors, associated with canonical pathways of iron homeostasis signaling, erythrocyte interaction with oxygen and carbon dioxide, erythropoietin signaling, heme biosynthesis, metabolism of porphyrins, and iron uptake and transport.

**Discussion:**

These findings describe significant differences in gene regulation during patient ICU course between survivors and non-survivors of COVID-19 ARDS. We identified multiple pathways that suggest heme and red blood cell metabolism contribute to disease outcomes. This approach is generalizable to larger cohorts and supports an approach of longitudinal sampling in ARDS molecular profiling studies, which may identify novel targetable pathways of injury and resolution.

## Introduction

From emergence to January 2024, coronavirus disease 2019 (COVID-19) has led to over 7 million deaths worldwide (1). Acute respiratory distress syndrome (ARDS) is one complication of severe COVID-19 and contribute to COVID-19 related death (2, 3). Among hospitalized patients with COVID-19, nearly one-third develop ARDS (4). Autopsy studies of COVID-19 reveal evidence of ARDS in most decedents (5, 6). Epidemiologic data from 2020 suggests at least a five-fold increase in ARDS-related deaths during the height of the COVID-19 pandemic (7). ARDS is a form of acute, non-cardiogenic, hypoxemic respiratory failure, characterized by bilateral lung infiltrates (8) that accounts for 10% of ICU admissions (9) with mortality rates ranging from around 30-50% (9). ARDS is notoriously heterogeneous, affecting varied patient populations with lung injury from various causes. One current focus of ARDS research is the identification of distinct patient subgroups that display varied outcomes and responses to targeted therapies (10–13). While clinical trials have demonstrated the efficacy of corticosteroids as treatment in COVID-19 ARDS (14, 15), studies evaluating immune modulating therapies in diverse ARDS populations have yielded mixed results (16, 17). Despite decades of clinical trials in ARDS, supportive care remains the primary treatment approach in diverse ARDS populations (18), and effective, targeted therapeutics for ARDS are lacking.

A better understanding of ARDS pathobiology may inform the limitations of current treatments, and the COVID-19 global pandemic has renewed the importance and urgency of new approaches to ARDS research. Recent studies have employed molecular profiling tools, such as RNA-sequencing, single-cell RNA-seq, and proteomics, with varied study design to better understand gene signatures that correlate with disease severity among COVID-19 ARDS patients. In this respect, multiple studies have highlighted the importance of type I interferon signaling and acute pro-inflammatory mediators in peripheral blood mononuclear cells (PBMCs) collected from patients early in disease course that correlate with COVID-19 disease severity (19–21), suggesting that PBMC analysis provides valuable insight into the dysregulated biology of COVID-19. As COVID-19 is dynamic with an evolving disease course over hours and days, other groups have examined transcriptomic data from patients at multiple time-points with varied sampling schema, including defined clinical stages (treatment, convalescence, and rehabilitation) (20), early and late ICU time points (22), and sampling from admission through two months of follow-up (23). Optimal strategies for patient sampling during the course of COVID-19 ARDS illness and recovery remain unclear. Our approach of longitudinal patient sampling provides opportunities to identify mechanisms that may contribute to disease progression or resolution.

To better understand how dynamic gene expression correlates with clinical outcomes among a group of patients with COVID-19 ARDS, we performed RNA-sequencing of patient PBMCs collected at 4 fixed time intervals across ICU admission. We hypothesized that longitudinal analysis may identify distinct transcriptomic changes compared to analysis at single time points to better characterize dynamic processes of injury and repair in COVID-19 ARDS. Here, we established three groups for comparison, including patients with COVID-19 ARDS that survived hospitalization, patients with fatal COVID-19 ARDS, and ARDS patients without COVID-19 as a control comparison group. We analyzed differential gene expression at each individual time point and performed longitudinal analysis to identify unique patterns of dynamic gene expression throughout acute illness.

## Methods

### Study design and identification of patient cohort

**Figure 1:**
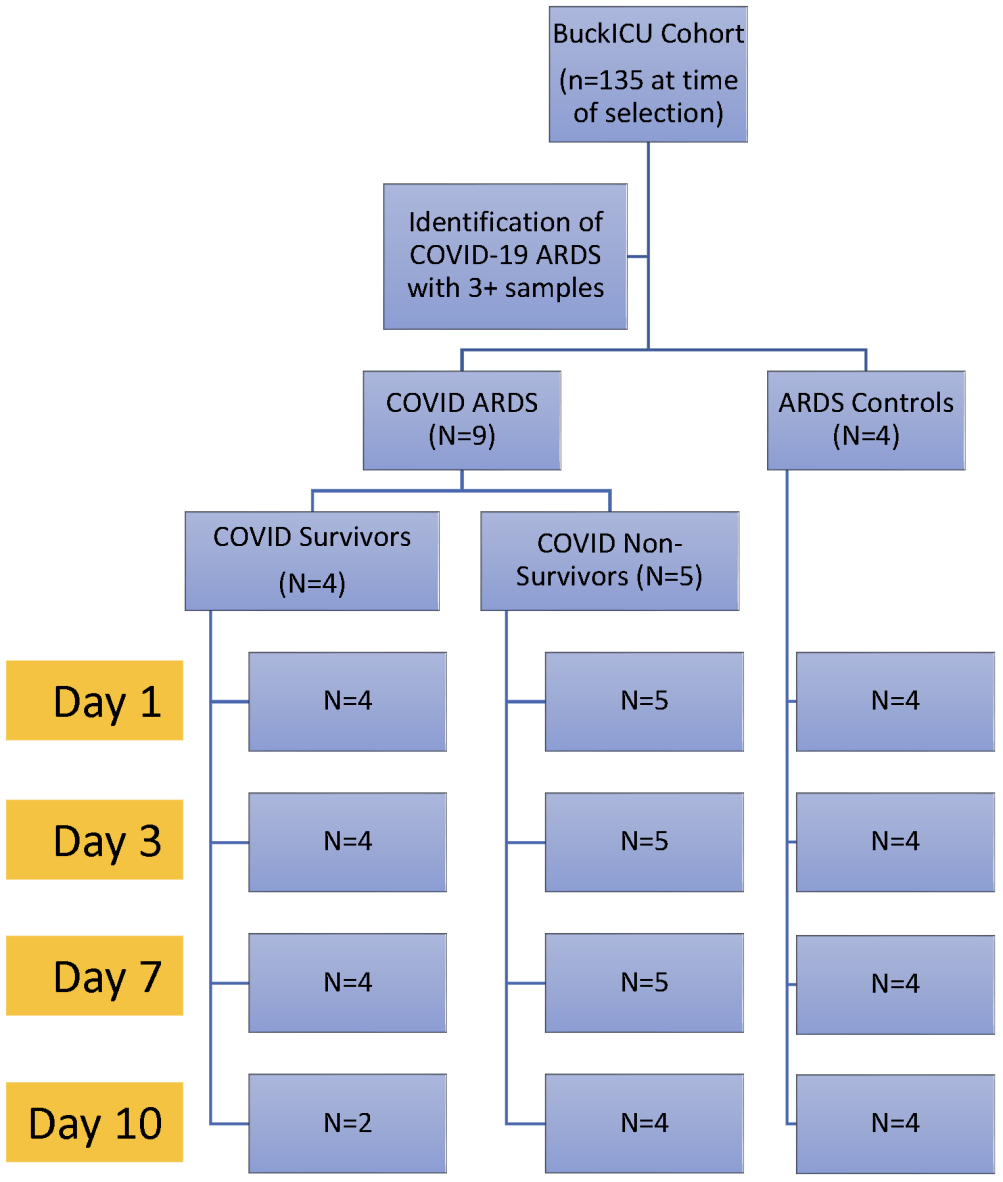
Experimental design. Patients were identified from the Ohio State University Intensive Care Unit Registry (BuckICU). Subjects with available longitudinal biosamples with COVID-19 ARDS or non-COVID-19 ARDS controls were selected for inclusion in the study.

Patients with ARDS with available longitudinal peripheral blood samples were identified from the Ohio State University Intensive Care Unit Registry (BuckICU), a pre-existing, IRB-approved (IRB #2020H0175) biorepository that enrolls patients within 48 hours of admission to the intensive care units at the Ohio State University Wexner Medical Center and the Arthur G. James Cancer Hospital and Richard J. Solove Research Institute with acute respiratory failure and/or suspicion of sepsis. For inclusion in the BuckICU biorepository, acute respiratory failure is defined by an increase in supplemental oxygen requirement to maintain oxygen saturation (SpO2) greater than 92% or the need for adjunctive respiratory support, including high flow nasal cannula, non-invasive positive pressure ventilation or mechanical ventilation. To screen patients for enrollment, BuckICU defines suspicion of sepsis as meeting SIRS criteria ((any two or more of White Blood Cell count > 12 or < 4 x 10^9^/L, heart rate > 90 beats per minute, respiratory rate > 20 breaths per minute, or temperature > 38°C or < 36°C) and clinical suspicion of infection (collection of any clinical culture specimen OR initiation of antibiotics)) (24). Following completion of the study protocol, patient cases are adjudicated by two pulmonary and critical care physicians. While SIRS criteria were used for screening purposes, Sepsis-3 guidelines were used to define sepsis during case adjudication. Sepsis-3 defines sepsis as organ dysfunction caused by dysregulated host response to infection represented by an increase in Sequential Organ Failure Assessment (SOFA) score of at least 2 points due to infection (25, 26). For this study, we identified patients between May 2020 and June 2021 who required mechanical ventilation at ICU admission with ARDS as defined by the Berlin definition (8), with at least 3 available longitudinal blood samples (days 1, 3, and 7). As this study aimed to identify differences in gene expression with correlation to COVID-19 ARDS mortality, we selected 9 patients with ARDS and positive SARS-CoV-2 upper respiratory tract testing by PCR and an additional 4 patients with ARDS and negative SARS-CoV-2 testing to characterize differences in gene expression that may be specific to COVID-19 status. As shown in **Figure 1**, we defined 3 groups: 1) patients with COVID-19 ARDS who survived hospitalization (COVID survivors, n = 4); 2) patients with COVID-19 ARDS who did not survive hospitalization (COVID non-survivors, n = 5); and 3) patients with ARDS from other causes (ARDS controls, n = 4).

### RNA extraction, RNA-seq library construction and sequencing

For gene expression profiling, we used longitudinal, banked, peripheral blood mononuclear cells (PBMCs), isolated by Ficoll (Sigma, Cytiva 17-1440-03) density gradient centrifugation of whole blood, lysed in Trizol (Invitrogen, 15596026), and stored at -80°C. We extracted RNA using the Direct-zol RNA Miniprep Plus kit (Zymo Research, R2071) followed by RNA cleanup with the Monarch RNA Cleanup Kit (New England BioLabs, #T2040, 50 μg) with additional in tube DNaseI treatment, per the manufacturer’s protocols. Total RNA was quantified using the Invitrogen Qubit RNA HS Assay kit (Invitrogen, Carlsbad, CA) and RNA quality was assessed by RNA integrity scoring using Agilent 2100 Bioanalyzer and/or 2200 TapeStation (Agilent, Santa Clara CA). RNA seq libraries were prepared using kits from New England Biolabs (Ipswich, MA) with 100 ng total RNA by targeted depletion of rRNA (NEB E#E6310x). Fragmentation and amplification were done using NEBNext Ultra II Directional (stranded) RNA Library Prep Kit (NEB#E7760L) and NEBNext (E64490S/L). Samples were sequenced to a depth of 40 million paired-end 150 bp clusters on the Illumina NovaSeq platform (Illumina, Inc., San Diego, CA) through the Ohio State University Genomics Shared Resource.

### Sequence alignment, gene count generation, and differential expression analysis

RNA sequencing sample reads underwent pre-alignment quality control with fastqc followed by alignment to the human genome GRCh38.96 (GCF_000001405.26) using HiSAT2 (v2.1.0) to obtain feature counts for each sample. Read alignment was performed through the Ohio Supercomputer Center (27). These counts were used for Differential Expression Analysis using the DESeq2 package in R (version 4.3.2). Lowly expressed genes were removed, and counts underwent normalization prior to differential expression analysis. Data visualizations were performed in R. We used the likelihood ratio test (LRT) to compare groups with or without time as an interaction term. Genes were determined to be significantly expressed based on adjusted *p* < 0.05 (false discovery rate). A variance stabilizing transformation (VST) was performed for normalization to create heat maps or cluster diagrams.

### Ingenuity pathway analysis

We utilized Ingenuity Pathway Analysis (IPA v23.0, Qiagen) software to analyze the resultant gene expression data to identify canonical pathways common to the DEGs in each comparison. DESeq2 results were uploaded to IPA along with their corresponding adjusted p values, as the LRT does not provide fold change results. Pathways were filtered for significance at a -log10 *p* value of 1.3, which corresponds to an FDR of 0.05.

## Results

### Subject cohort characteristics

Subject characteristics are shown in **Table 1**. Per group definitions, all subjects in the COVID non-survivors group did not survive hospitalization, while all subjects in the COVID survivors group survived critical illness. All patients met ARDS criteria during ICU admission, required mechanical ventilation with low tidal volume ventilation, and received standard ICU supportive care. At Day 1, P/F ratios were similar across groups. Subjects with COVID-19 showed decreased P/F at Day 3, compared to Day 1, and COVID-19 survivors showed improvements in P/F later in ICU admission, compared to COVID-19 non-survivors. ARDS controls and COVID-19 non-survivors had significantly higher SOFA scores at Day 1 with higher SOFA scores throughout ICU admission compared to COVID-19 survivors. Further, vasopressor requirements were higher in the ARDS controls and COVID-19 non-survivors compared to COVID-19 survivors. All ARDS controls had sepsis from a bacterial infection as a risk factor leading to ARDS. These patients received treatment per current sepsis care guidelines (28, 29), including source control, early antibiotics, and volume resuscitation. All COVID-19 subjects tested positive for SARS-CoV-2 by upper respiratory tract nucleic acid amplification test, and all subjects were unvaccinated against SARS-CoV-2. Some COVID-19 patients had positive bacterial cultures during the study period, but COVID-19 was determined to be the primary risk factor for ARDS. There were differences in steroid exposure among groups, as all patients in the COVID-19 survivors group received dexamethasone, while 4 of 5 COVID-19 non-survivors and no ARDS Controls received dexamethasone.

**Table 1:** Values shown as median (range) or percentage of subjects.

Characteristics	ARDS Controls	COVID-19 Non-Survivors	COVID-19 Survivors	P (all groups)
N	4	5	4	
Age	70 (52-83)	70 (61-84)	59 (34-75)	0.3
Male Sex	50%	80%	75%	0.8
White Race	100%	80%	50%	0.5
In Hospital Mortality Time to Mortality (days)	25% 23 (23)	100% 18.4 (10 – 23)	0%	0.005
ARDS	100%	100%	100%	
P/F ratio				
Day 1	127 (78 – 181)	114 (46 – 147)	122 (70 – 203)	0.74
Day 3	133 (118 – 182)	98 (67 – 136)	103 (84 – 203)	0.13
Day 7	137 (107 – 153)	123 (86 – 162)	171 (159 – 203)	0.06
Day 10	136 (101 – 213)	133 (103 – 223)	200 (196 – 203)	0.67
Day 1 WBC	24 (12-39)	9 (6-10)	8 (3-16)	0.038
Vasopressors	50%	40%	0%	0.2
SOFA Score				
Day 1	12 (9-17)	6 (5-13)	3.5 (2-8)	0.03
Day 3	8.5 (8-20)	10 (5-14)	4 (2-8)	0.08
Day 7	5.5 (5-16)	10 (6-12)	4 (2-8)	0.11
Day 10	6 (5-13)	11 (5-15)	4.5 (3-6)	0.26
Sepsis	100%	100%	100%	
Bacterial Infection	100%	60%	25%	
Blood	25%	40%	0%	
Lung	75%	40%	25%	
Urinary	25%	20%	0%	
SARS-CoV-2 Positive	0%	100%	100%	
Dexamethasone	0%	80%	100%	0.015
Remdesivir	0%	60%	100%	0.023
Convalescent Plasma	0%	40%	75%	0.15

P-values determined by Fisher’s exact test or Kruskal -Wallis rank sum test. Time to mortality is defined as interval from Day 1 sample to date of death. Sepsis was defined by Sepsis-3 guidelines. Sepsis from bacterial infection was determined to be the primary risk factor for ARDS in the ARDS control group.

### Analysis of differential gene expression at single time points highlights heterogeneity of host responses during ARDS

**Figure 2:**
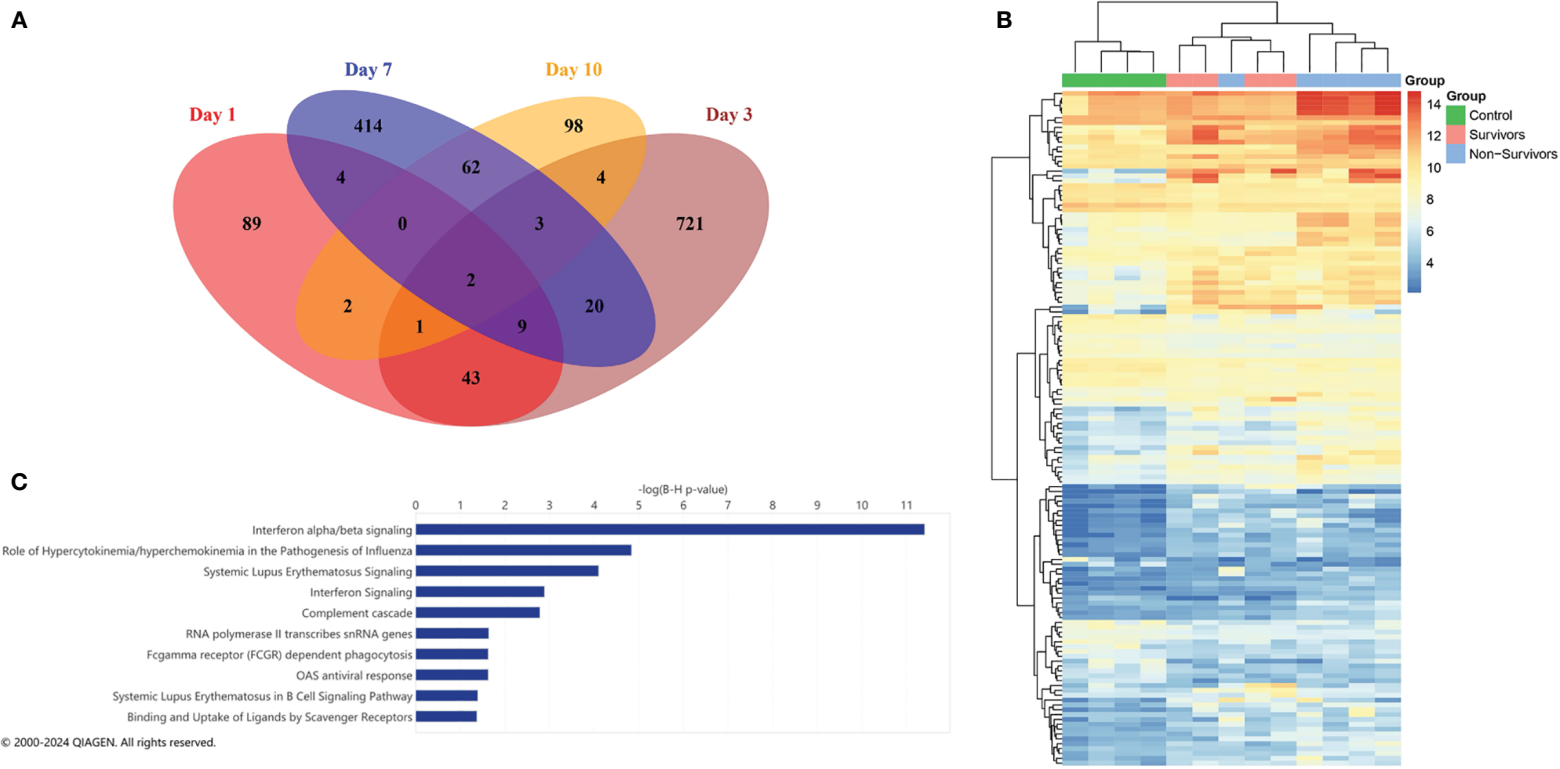
Differential expression analysis on individual days. **(A)** Venn diagram showing overlap of significant differentially expressed genes for each day. **(B)** Heatmap of the 150 significant DEGs (FDR < 0.05) identified at Day 1 with column clustering by gene expression pattern. **(C)** Ingenuity pathway analysis (IPA) of canonical pathways identified by DEGs at Day 1. The length of the bar indicates statistical significance of each pathway using -log10 BH multiple correction p-value.

Prior to analyzing dynamic gene expression patterns across ICU admission, we first performed differential expression analysis at each time point (days 1, 3, 7, and 10), comparing the three groups (COVID survivors, COVID non-survivors, and ARDS controls). Prior studies had analyzed transcriptomes of biospecimens from early time points of severe COVID-19 and identified genes associated with interferon signaling, T cell activation, and acute inflammation that correlated with worse outcomes. Comparing our 3 groups at each collection day, we identified 150 differentially expressed genes (DEGs) at Day 1, 803 DEGs at Day 3, 514 DEGs at Day 7, and 172 DEGs at Day 10. We next identified common differences in gene expression across time points by comparing the lists of DEGs and observed that the majority of DEGs identified at each timepoint were unique (**Figure 2A**). For example, differences in gene expression profiles peaked at Day 3. However, of the 803 DEGs identified at Day 3, only 82 of these transcripts were determined to be significantly different at the other time points. We observed greatest overlap between Days 1 and 3 (43 common DEGs) and Days 7 and 10 (62 common DEGs).

As prior studies have characterized differential expression at early time points in COVID-19, we further examined the 150 DEGs identified at Day 1. The top 20 DEGs identified at Day 1 between the three groups are shown in **Table 2**. Clustering of DEGs revealed correlation with our pre-defined comparison groups apart from one non-survivor, who displayed a pattern of expression similar to COVID survivors (**Figure 2B**). Considering all COVID-19 patients, we observed increases in interferon-stimulated genes, including interferon alpha-inducible protein 6 (IFI6), interferon alpha-inducible protein 27, mitochondrial (IFI27) (**Table 2**). Ingenuity pathway analysis (IPA) of DEGs revealed canonical pathways primarily related to interferon signaling (**Figure 2C**).

**Table 2:** Top 20 most significant DEGs identified at Day 1.

Ensemble ID	Gene Symbol	Entrez Gene Name	Adjusted P-value
ENSG00000160932	LY6E	lymphocyte antigen 6 family member E	3.47E-04
ENSG00000207389	RNU1-4	RNA, U1 small nuclear 4	6.21E-04
ENSG00000273768	LOC124904613	U1 spliceosomal RNA	7.50E-04
ENSG00000200795	RNU4-1	RNA, U4 small nuclear 1	7.50E-04
ENSG00000126709	IFI6	interferon alpha inducible protein 6	1.11E-03
ENSG00000228672	PROB1	proline rich basic protein 1	1.11E-03
ENSG00000202538	RNU4-2	RNA, U4 small nuclear 2	1.11E-03
ENSG00000075142	SRI	sorcin	1.11E-03
ENSG00000252947	SCARNA1	small Cajal body-specific RNA 1	1.41E-03
ENSG00000211658	IGLV3-27	immunoglobulin lambda variable 3-27	1.56E-03
ENSG00000223416	RPS26P15	ribosomal protein S26 pseudogene 15	1.85E-03
ENSG00000207513	RNU1-3		1.89E-03
ENSG00000206652	RNU1-1	RNA, U1 small nuclear 1	1.93E-03
ENSG00000238025	ZDHHC4P1	zinc finger DHHC-type containing 4 pseudogene 1	2.27E-03
ENSG00000206596	RNU1-27P	RNA, U1 small nuclear 27, pseudogene	2.31E-03
ENSG00000275405	LOC124905321	U1 spliceosomal RNA	2.33E-03
ENSG00000264940	SNORD3C	small nucleolar RNA, C/D box 3C	2.75E-03
ENSG00000206585	RNVU1-7	RNA, variant U1 small nuclear 7	2.98E-03
ENSG00000265185	SNORD3B-1	small nucleolar RNA, C/D box 3B-1	3.24E-03
ENSG00000207005	RNU1-2		3.63E-03

IPA was used for annotation.

### Longitudinal analysis of gene expression reveals additional host response mechanisms

**Figure 3:**
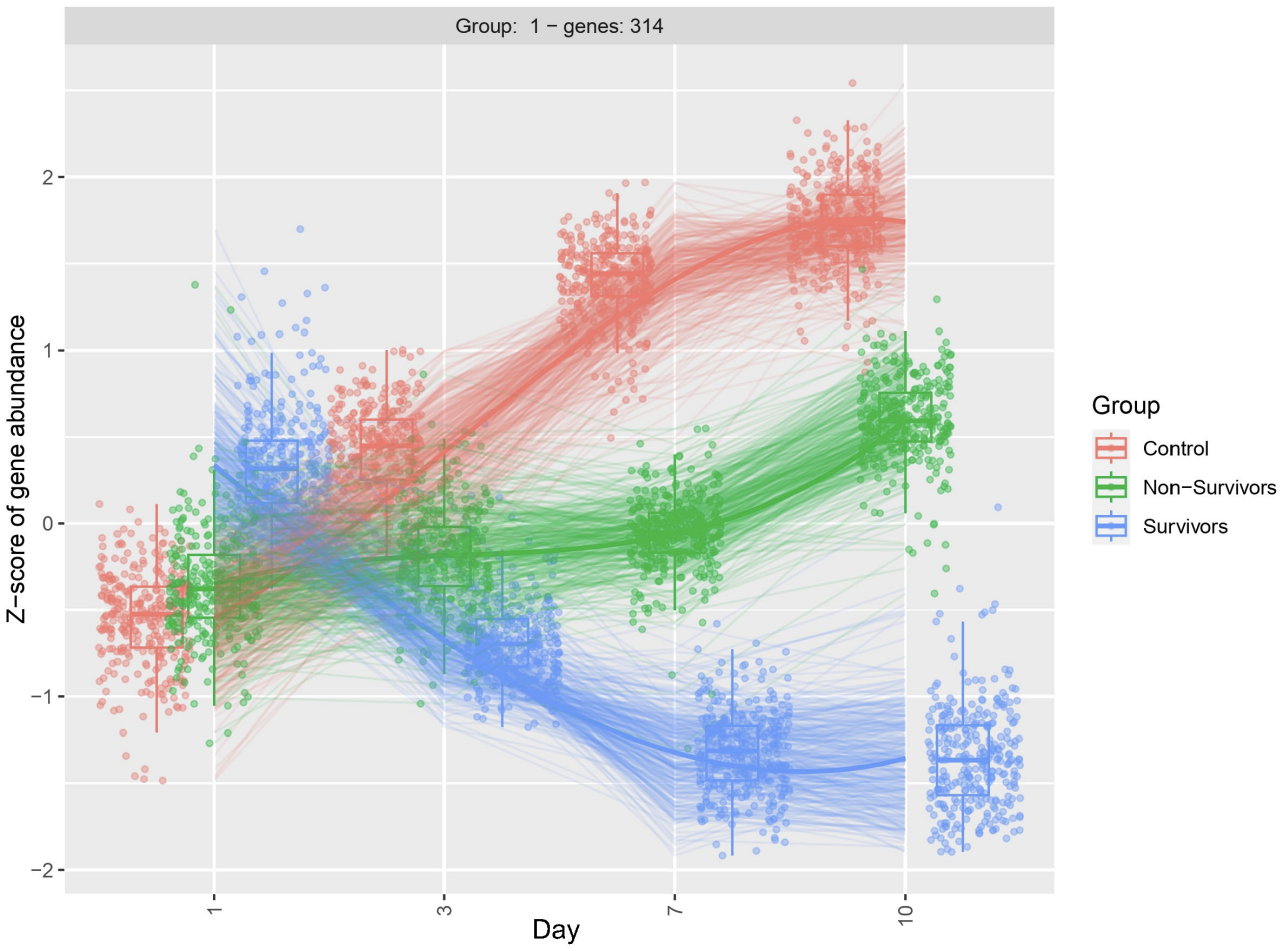
Clustering diagram demonstrates differential dynamic gene expression across time. Using the likelihood ratio test, we compared differences in dynamic gene expression across 3 patient groups, COVID-19 ARDS survivors, COVID-19 ARDS non-survivors, and ARDS controls and 4 time points with Day 1 as reference. DEGs were clustered by similar patterns of gene expression. 314 of the 341 DEGs identified by the longitudinal analysis showed a similar pattern of dynamic change. Significance determined by the adjusted p-value from DESeq2 analysis.

As we observed significant heterogeneity of differential gene expression at each time point, we next compared dynamic gene expression across time. As opposed to identifying differences at one timepoint, this approach allows for identification of transcripts that show differential patterns of expression across time (i.e. Which genes change over time? Are those patterns of change different between groups)? Here we identified 341 genes with significant differential expression across timepoints (p < 0.05). The top 20 significant DEGs identified by our longitudinal analysis are shown in **Table 3**. Notably, this approach only determines significance and does not specify fold change. After filtering for significant DEGs, we next performed additional visualizations to determine directionality and patterns of change. First, clustering analysis identified transcripts that demonstrate similar patterns of temporal change. Of the 341 DEGs, 314 showed similar temporal patterns (**Figure 3**). The top 20 genes demonstrating the most significant differences include hemoglobin subunit alpha 2 (HBA1, HBA2), hemoglobin subunit beta (HBB), von Willebrand factor C and EGF domains (VWCE), and carbonic anhydrase 1 (CA1) (**Table 3**). Among COVID survivors, the 314 genes showed downregulation of expression over time with a nadir by Day 7, compared to the COVID non-survivors and ARDS controls. The ARDS control patients demonstrated a pattern of increase in temporal gene expression, and the COVID non-survivors demonstrated delayed increases in these significantly changed genes over time (**Figure 3**).

**Table 3:** Top 20 most significant DEGs identified by longitudinal analysis.

Ensemble ID	Gene Symbol	Entrez Gene Name	Adjusted P-value
ENSG00000206172	HBA1/HBA2	hemoglobin subunit alpha 2	1.58E-05
ENSG00000167992	VWCE	von Willebrand factor C and EGF domains	1.58E-05
ENSG00000244734	HBB	hemoglobin subunit beta	2.09E-05
ENSG00000188536	HBA1/HBA2	hemoglobin subunit alpha 2	4.94E-05
ENSG00000158578	ALAS2	5’-aminolevulinate synthase 2	6.33E-05
ENSG00000013306	SLC25A39	solute carrier family 25 member 39	1.65E-04
ENSG00000153574	RPIA	ribose 5-phosphate isomerase A	2.69E-04
ENSG00000103342	GSPT1	G1 to S phase transition 1	3.85E-04
ENSG00000136732	GYPC	glycophorin C (Gerbich blood group)	3.85E-04
ENSG00000198176	TFDP1	transcription factor Dp-1	3.85E-04
ENSG00000158856	DMTN	dematin actin binding protein	3.89E-04
ENSG00000105701	FKBP8	FKBP prolyl isomerase 8	3.89E-04
ENSG00000159335	PTMS	parathymosin	3.89E-04
ENSG00000143774	GUK1	guanylate kinase 1	4.02E-04
ENSG00000107262	BAG1	BAG cochaperone 1	4.92E-04
ENSG00000133742	CA1	carbonic anhydrase 1	4.92E-04
ENSG00000167671	UBXN6	UBX domain protein 6	4.92E-04
ENSG00000124098	FAM210B	family with sequence similarity 210 member B	5.00E-04
ENSG00000099804	CDC34	cell division cycle 34, ubiquitin conjugating enzyme	5.87E-04
ENSG00000060138	YBX3	Y-box binding protein 3	6.12E-04

IPA was used for annotation.

**Figure 4:**
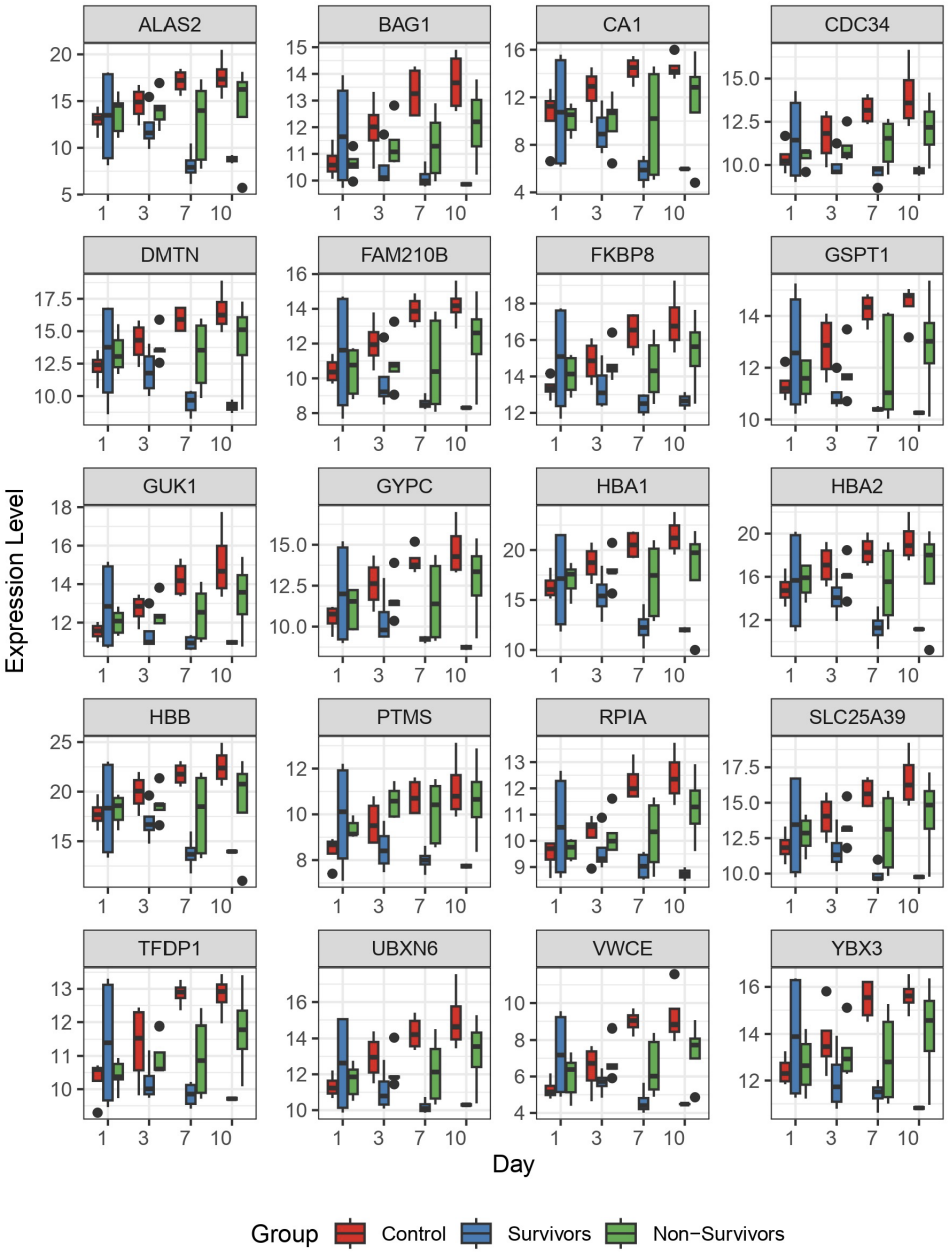
The top 20 differentially expressed genes identified by longitudinal analysis. Following identification of significantly differentially expressed genes by longitudinal analysis, we plotted the 20 genes with most significant differences to observe individual patterns of change. Here, we plotted normalized gene expression by variance stabilizing transformation (y-axis) against time (days 1, 3, 7, and 10 on the x-axis). Colored boxes represent the 3 comparison groups.

To better understand dynamic gene expression at the level of individual genes, we used box plots to visualize gene expression of the top 20 genes identified by the LRT. As expected, these genes demonstrate a similar pattern of change that was observed in the clustering analysis (**Figure 3**). Specifically, we observe the most variability in gene expression at day 1 for the COVID survivors, compared to the other two comparison groups. Further, differences in gene expression between groups increases across time (**Figure 4**). Together, these findings suggest that differences in host response across disease course may better inform underlying biological processes contributing to outcomes than differential gene expression at ICU admission.

### Perturbations in hematopoiesis and erythrocyte function pathways correlated with COVID-19 fatality

**Figure 5:**
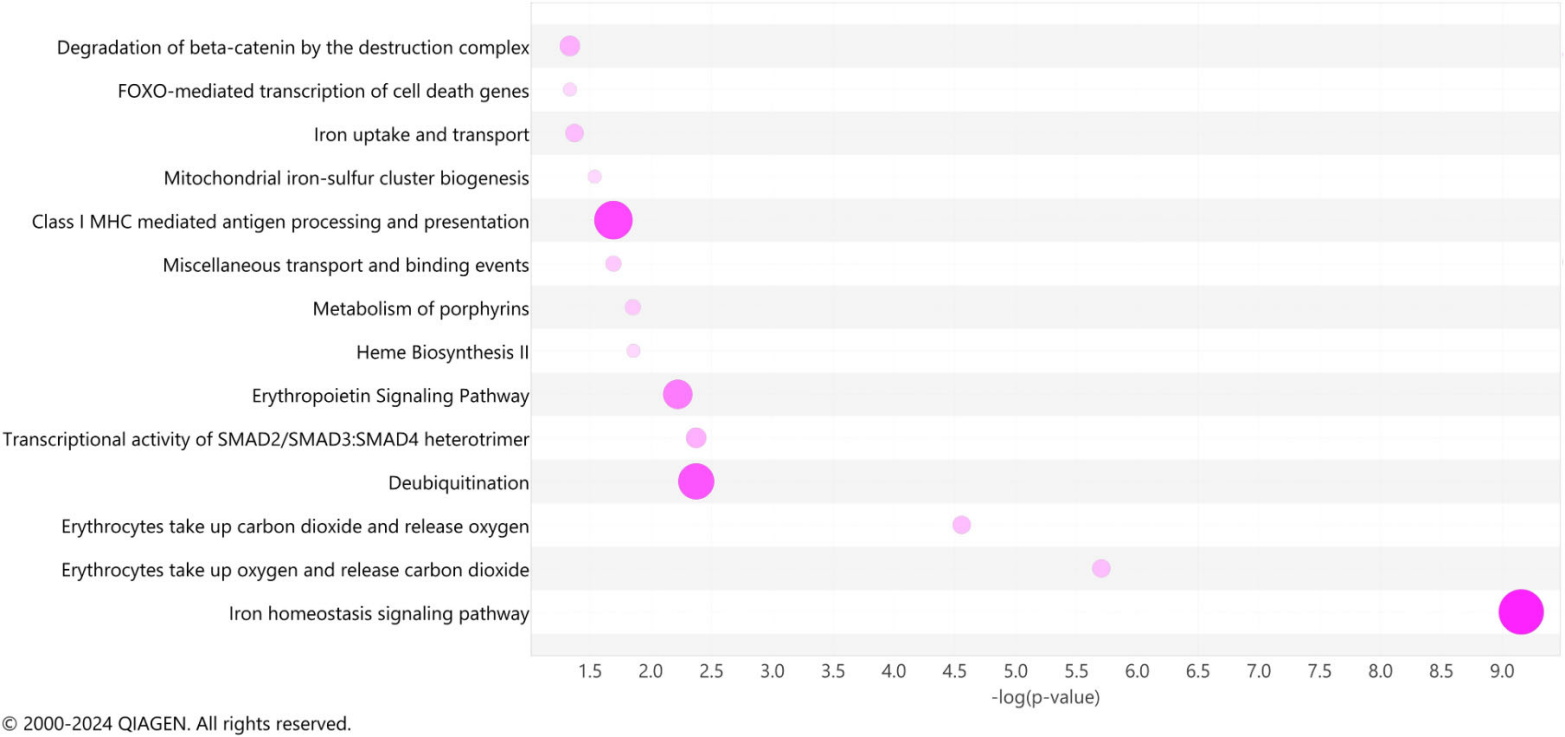
Ingenuity pathway analysis. A bubble chart shows the top pathway categories of the IPA canonical pathways of significant genes (adjusted p < 0.05) detected across all days simultaneously. The size and color intensity of bubbles indicates the number of genes overlapping each pathway.

To identify common biological pathways among the significant DEGs observed in our LRT comparisons, we utilized Ingenuity Pathway Analysis (IPA) to elucidate the pathways engaged among COVID survivors, non-survivors, and ARDS controls. As we compared 3 groups, this analysis does not specify fold-change differences or directionality and describes only significance and pathway enrichment, determined by the number of DEGs involved in each pathway. Among the most represented pathways, we identified iron homeostasis signaling, erythrocyte interaction with oxygen and carbon dioxide, transcriptional activity of SMAD2/SMAD3:SMAD4 heterotrimer, deubiquitination, erythropoietin signaling, heme biosynthesis, metabolism of porphyrins, and iron uptake and transport among the top 12 IPA canonical pathways (**Figure 5**). These results suggest that oxygen carrying capacity and metabolism of heme may be important modulators of disease course in COVID-19 ARDS.

## Discussion

Here we performed longitudinal RNA sequencing analysis of PBMCs from patients with ARDS at a single center during the COVID-19 pandemic (May 2020 – June 2021) and compared temporal gene expression changes between COVID-19 ARDS survivors and non-survivors, as well as non-COVID ARDS patients, across 10 days of ICU admission. Longitudinal analysis revealed 341 transcripts with significantly different patterns of dynamic gene expression over time with most significant differences in pathways of iron homeostasis, heme biosynthesis, and erythrocyte function, that remained upregulated throughout ICU course in fatal COVID-19 ARDS compared to survivors of COVID-19 ARDS. Enriched gene signatures of hemoglobin metabolism have previously been described in blood leukocytes from septic patients (30). *In vitro* studies have shown induction of hemoglobin genes during cellular stress in murine macrophages (31) and human PBMCs (32). The role of heme in macrophages is complex (32–34) and additional data are needed to support strategies targeting heme biosynthesis in ARDS patients. Notably, our study employing longitudinal sampling and analysis identified distinct pathway regulation throughout disease course that was not identified at single time points. This study highlights the dynamic nature of COVID-19 ARDS and represents a novel approach towards better understanding of COVID-19 ARDS pathobiology.

The COVID-19 pandemic increased the global incidence of severe viral pneumonia, acute respiratory failure, and ARDS (7). Clinicians and researchers alike observed unique features of COVID-19 ARDS and raised the question: Is COVID-19 ARDS somehow different than “regular” ARDS (35, 36)? As a syndrome, ARDS is known for its heterogeneity and efforts to identify subtypes and subgroups of ARDS patients predate the pandemic (10, 13). Our study identified a cohort of ARDS patients with COVID-19 and varied clinical outcomes and compared to a group of patients with ARDS and bacterial sepsis as the primary ARDS risk factor. Our differential expression analysis on Day 1 of study enrollment was characterized by significant activation of immune system pathways and interferon signaling consistent with prior reports in studies of severe COVID-19 (37–40). Dysregulated interferon signaling has been implicated in COVID-19 as an important driver of pathology (21). Compared to healthy controls, interferon responses are elevated in COVID-19 (41). However, when compared to other viral infections or among COVID-19 patients with varied clinical course, interferon responses are more variable (42). Subjects in our comparison group of non-COVID-19 ARDS had sepsis from bacterial sources as their primary risk factor for ARDS. Host responses to bacterial versus viral infection yield different transcriptomic signatures (43–45), which may account for the significant differences in interferon responses in our analysis. While multiple interferon-stimulated genes showed significant upregulation in our analysis, one specific gene, interferon-inducible protein 27 (IFI27), showed significant upregulation at study enrollment in COVID-19 ARDS compared to non-COVID-19 ARDS. This recapitulates findings of other study that suggest robust and specific upregulation of IFI27 in COVID-19. A re-analysis of nine independent cohort studies that analyzed peripheral blood gene expression across multiple infections, including COVID-19, influenza, and bacterial pneumonia, described a COVID-19-specific gene signature comprised of 149 genes, among which IFI27 was the sole gene directly associated with the interferon response (46). A single-center cohort study comparing peripheral blood transcriptomes at one time point showed IFI27 was highly upregulated in COVID-19, even when compared to influenza and seasonal coronaviridae (42). Despite reports of varied interferon responses, robust induction of IFI27 may represent a peripheral blood gene signature unique to COVID-19 infection. Notably, our study, like the above referenced COVID-19 transcriptomics studies, focused on mixed populations of peripheral blood cells, which may reflect transcriptomic signatures driven by a single cell type or changes in relative cell populations. Further, PBMCs represent transcriptional signatures in peripheral blood, which may differ from the lung transcriptome (47).

Despite multiple prior reports of transcriptomics in COVID-19, our study is unique in our sampling and analysis strategy. In opposition to single time-point studies or limited longitudinal sampling, we analyzed peripheral blood gene expression throughout acute illness at multiple short intervals. While our study is limited by the small sample size and single-center design, it provides proof of concept that longitudinal molecular profiling can be valuable for identifying dynamic molecular mechanisms that function during ARDS disease course. In ARDS studies, analysis at individual timepoints allows for identification of relative differences in gene expression between groups. However, this design is vulnerable to baseline patient heterogeneity and lead-time bias. While longitudinal sampling is subject to the same obstacles, our approach allows each patient to function as their own baseline control and focuses on dynamic gene expression during acute illness. We identified gene signatures related to iron homeostasis, erythropoietin signaling, heme biosynthesis, and iron uptake that we grouped into processes contributing to erythropoiesis, as well as upregulation of erythrocyte function in fatal COVID-19 compared to COVID-19 survivors. Among ARDS controls, we observed early upregulation of these genes compared to the other groups with decreased rate of change between days 7 and 10. Among the COVID-19 non-survivors, gene expression related to erythropoiesis and erythrocyte function showed late increased compared to COVID-19 survivors, which showed relatively low expression throughout ICU course. Other groups have employed sampling at multiple time points with various strategies and have also identified gene expression correlating with pathways of hemopoiesis, reactive oxygen species, and erythrocyte functioning (20, 22, 23). Zheng et al. examined longitudinal transcriptomes but defined three *ad hoc* clinical stages (treatment, convalescence, and rehabilitation) as opposed to consistent time intervals; they identified early downregulation of genes related to humoral immunity and type I interferon response and upregulated gene expression related to hemopoiesis, regulation of inflammatory response, mRNA splicing via spliceosome, and epithelial cell proliferation during COVID-19 recovery (20). Another study applied longitudinal multi-omics with 2 – 7 times points from 0 – 55 days after admission and demonstrated increased protein catabolism, erythrocyte differentiation, ferroptosis, and organelle disassembly in clusters primarily corresponding to COVID non-survivors compared to COVID survivors (23). As our study and others have identified common pathways of iron homeostasis and erythrocyte function, it is intriguing to hypothesize that interventions targeting effective erythropoiesis, iron handling, and erythrocyte function represent a novel therapeutic strategy in COVID-19 ARDS. Indeed, studies have demonstrated perturbations in iron handling and ferroptosis (iron-dependent cell death) due to COVID-19 in humans (48), animal models (49), and human cells (50), suggesting that COVID-19 may uniquely impact iron homeostasis and downstream pathways during disease course. Further, observational human studies, including ours, may be capturing a compensatory mechanism of increased erythropoiesis and erythrocyte function in severe COVID-19. Steroid treatment with dexamethasone is a common and accepted treatment in severe COVID-19 (14, 15) and may impact iron metabolism pathways (51). In our cohort, steroid exposure was high in the COVID-19 groups, as 8 of 9 subjects received dexamethasone. However, ARDS controls did not receive steroid treatment. All patients in our cohort met sepsis criteria, recognizing the frequent overlap between critical illness syndromes, such as ARDS and sepsis. Among septic patients, heme metabolism and iron homeostasis genes are enriched in white blood cells (neutrophils and PBMCs) compared to controls (30), and transcriptomic differences in heme biosynthesis genes correlated with sepsis subgroups or endotypes (52). In non-erythroid cells, hemoglobin scavenges free radicals and functions in nitric oxide metabolism (53). Cellular damage through reactive oxygen species is a known pathologic mechanism in both sepsis and ARDS. Our study and others (23, 52) demonstrate increased heme-related transcript expression correlated with worse outcomes in certain critically ill populations. We postulate that upregulation of these transcripts in non-erythroid cells represents a compensatory mechanism due to ongoing oxidative stress. As iron and heme metabolism are foundational biologic processes, it is unclear if these pathways represent viable interventional targets during COVID-19 ARDS.

Our study supports a novel patient sampling strategy and demonstrates a unique analysis of dynamic gene expression. We focused on patients with severe COVID-19 ARDS as a subgroup of ARDS patient to gain insights into disease pathology. Our findings suggest an approach for future studies, generalizable to larger COVID-19 and ARDS cohorts. Our study is limited by several factors. First, all patients were enrolled from a single center for analysis, which may not be representative of the general population. As we analyzed multiple samples from each subject, the sample size of the cohort was small. While we attempted to select age- and sex-matched subjects, the sample size does not allow for proper control of these potentially confounding variables. Despite the small cohort size, we were able to identify several biologically relative pathways, reflected in correlation with prior studies, and provide new insights via our longitudinal approach. We defined outcomes by mortality in this study. While our COVID-19 ARDS non-survivors demonstrate impaired oxygenation throughout ICU stay, consistent with worsened ARDS, this group also had baseline higher SOFA scores that increased over time and increased vasopressor requirements, which may confound our findings. Further, this study used ARDS as a primary inclusion criterion, but severe COVID-19 is a systemic disease. All patients in our cohort met criteria for sepsis, and we did not attempt to differentiate COVID-19 ARDS, sepsis, or both as independent groups. Our transcriptomic findings in peripheral blood cells may be related to systemic responses instead of lung-specific pathology related to COVID-19 or ARDS. Disease classification in critical illness is currently limited by often overlapping syndrome-based paradigms (54, 55). However, transcriptomic studies have the potential to identify dysregulated host response pathways during critical illness that inform clinical disease course beyond established definitions of ARDS or sepsis (56).

## Conclusion

Patients with ARDS from COVID-19 may represent a subgroup of ARDS patients with distinct molecular pathophysiology that drive disease outcomes. Our study identified differences in dynamic expression of genes related to iron homeostasis and erythrocyte function that correlate to survival in COVID-19 ARDS. Our findings are supported by prior studies of molecular profiling in COVID-19 and suggest that iron handling and ferroptosis may be putative mechanisms of ongoing lung injury during SARS-CoV-2 infection. Additional research is needed to examine the therapeutic potential of these pathways. Finally, our study suggests that short interval longitudinal sampling during acute illness may uncover novel mechanisms of injury, repair, and resolution during COVID-19 ARDS. This approach should be considered further for future ARDS molecular profiling study design.

## Data availability statement

The data presented in the study are deposited in the Gene Expression Omnibus (https://www.ncbi.nlm.nih.gov/geo/), accession number GSE273149.

## Ethics statement

The studies involving humans were approved by The Ohio State University Biomedical Sciences Institutional Review Board. The studies were conducted in accordance with the local legislation and institutional requirements. The human samples used in this study were acquired from an existing biorepository that collects and stores human biosamples and data from critically-ill patients at Ohio State University. The biorepository project utilizes broad consent to collect and store samples for use in secondary analyses. Written informed consent for participation was not required from the participants or the participants’ legal guardians/next of kin in accordance with the national legislation and institutional requirements.

## Author contributions

ME: Formal Analysis, Validation, Visualization, Writing – original draft, Writing – review & editing, Data curation, Investigation. FJ: Data curation, Investigation, Writing – review & editing, Methodology. DF: Investigation, Methodology, Writing – review & editing, Supervision. LL: Investigation, Writing – review & editing, Data curation. SC: Data curation, Investigation, Writing – review & editing. KH: Data curation, Investigation, Writing – review & editing. SK: Data curation, Investigation, Writing – review & editing. GS: Data curation, Investigation, Writing – review & editing. SP: Data curation, Investigation, Writing – review & editing, Conceptualization, Methodology. JH: Methodology, Writing – review & editing, Funding acquisition, Project administration, Resources, Supervision. RM: Writing – review & editing, Funding acquisition, Methodology, Project administration, Resources, Supervision. JE: Methodology, Writing – review & editing, Data curation, Investigation, Writing – original draft. JB: Methodology, Writing – original draft, Writing – review & editing, Conceptualization, Formal Analysis, Funding acquisition, Project administration, Visualization.
